# Profiling of CYP4Z1 and CYP1B1 expression in bladder cancers

**DOI:** 10.1038/s41598-021-85188-4

**Published:** 2021-03-10

**Authors:** Yousef M. Al-saraireh, Fatemah O. F. O. Alshammari, Ahmed M. M. Youssef, Sameeh Al-Sarayreh, Ghadeer H. Almuhaisen, Nedal Alnawaiseh, Jehad M. Al Shuneigat, Hamzeh M. Alrawashdeh

**Affiliations:** 1 Department of Pharmacology, Faculty of Medicine, Mutah University, P.O.Box 7, Karak, 61710 Jordan; 2 Department of Medical Lab Technology, Faculty of Health Sciences, The Public Authority for Applied Education and Training, P.O.Box 14281, 15432 Shuwaikh, Kuwait; 3 Department of Pharmacology, Faculty of Pharmacy, Mutah University, P.O.Box 7, Karak, 61710 Jordan; 4 Department of Biochemistry and Molecular Biology, Faculty of Medicine, Mutah University, PO. Box 7, Karak, 61710 Jordan; 5 Department of Microbiology and Pathology, Faculty of Medicine, Mutah University, P.O. Box 7, Karak, 61710 Jordan; 6 Department of Public Health, Faculty of Medicine, Mutah University, P.O. Box 7, Karak, 61710 Jordan; 7 Department of Ophthalmology, Ibn Al Haytham Hospital, P.O.Box 410739, Amman, Jordan

**Keywords:** Biological techniques, Cancer, Molecular medicine, Oncology

## Abstract

Bladder cancer is the tenth most common cancer worldwide, where its burden remains a challenge and needs new novel therapies. Several reports indicate expression of CYP4Z1 and CYP1B1 in many tumours. Their expressions are associated with a poor prognosis, and therefore proposed as promising biomarkers or targets for anticancer therapy. By using immunohistochemistry, expression of CYP4Z1 and CYP1B1 was evaluated in a panel of different types of bladder cancer, and the enzymes’ relation to histopathological features were assessed. Results showed an increased expression of CYP4Z1 (54.3%) and CYP1B1 (76.9%) in the majority of bladder cancers compared to weak or lack of expression of both enzymes in normal tissues. CYP4Z1expression was significantly associated with tumour grade and stage where the expression was markedly increased in a high grade and advanced stage of the disease (*p* < 0.05). Additionally, CYP1B1 expression was also associated with TNM staging (*p* < 0.05) and its expression was increased in patients with lymph node metastasis. The expression profiles of CYP4Z1 and CYP1B1 suggest that both enzymes have the potential to be biomarkers or targets for novel anticancer therapy for bladder cancer. Nevertheless, further studies are needed to better delineate whether these enzymes are druggable targets.

## Introduction

Bladder cancer is the most common cancer in industrialized regions. It accounts for roughly 3% of cancers worldwide. It is the tenth most prevalent cancer and the thirteenth most deadly cancer worldwide^1^. It is estimated that 550,000 (3%) people were diagnosed and 200,000 (2.1%) died from bladder cancer in 2018^2^. Moreover, its incidence is roughly four-fold more prevalent in men than women^1^. This gender discrepancy in the incidence of bladder cancer is attributed to the gender disparity in smoking status, sex steroid hormone pathway, occupational exposure, metabolic enzyme activity^3^. Histopathologically, 90% of bladder cancer cases arise from urothelial cells while the remaining 10% falls into other subtypes such as adenocarcinomas and squamous cell bladder cancers^1^. This cancer is highly associated with smoking tobacco, occupational exposure to carcinogens, schistosomiasis and contaminants in drinking water. Recently, the overall survival rate of bladder cancer patients has improved with early diagnosis, robotic surgical techniques, and the introduction of immunotherapy^4^. However, bladder cancer burden remains a challenge and new therapies for bladder cancer are urgently needed.

Nowadays, many metabolic enzymes are currently under examination for their potential roles in bladder cancer susceptibility. Of these are members of the cytochrome P450 (CYP) family, CYP4Z1 and CYP1B1.

Many studies reported selective expression of several forms of CYP4 in cancers. Of recent focus is the aberrant expression of CYP4Z1 in many cancers. This orphan enzyme was selectively expressed in cancers of the breast, ovary and prostate compared with healthy controls^5–10^. Its overexpression was associated with poor patient outcome and proposed as a biomarker for malignancy or progression of CYP4Z1 expressing cancers^7,8^. Moreover, the expression was abnormally localized to the cell surface of breast cancer cells and led to the production of CYP4Z1 autoantibodies that may serve as a biomarker for breast cancer progression^11^. Importantly, data from the Human Protein Atlas on profiling of mRNA levels of CYP4Z1 in 406 patients with urothelial cancer showed that 115 (28.3%) patients had high expression, where as other patients (291, 71.7%) demonstrated low expression^12^. Using cell lines models, CYP4Z1 expression enhanced tumour growth, angiogenesis and the spread of tumour cells in both in vitro and in vivo models. These effects were due to decrease in the levels of myristic and lauric acids and the conversion of arachidonic acid to 20-hydroxyeicosatetraenoic acid (20-HETE) by CYP4Z1^13^. The tendency of CYP4Z1 to metabolize these particular endobiotics has been reported. CYP4Z1 was found to mediate ω-hydroxylation of myristic and lauric acids producing different ω-hydroxylated metabolites and arachidonic acid to 20-HETE^13,14^. However, a recent investigation contrasts with the hypothesis where 20-HETE metabolites were absent in the CYP4Z1 reaction with arachidonic acid and presence of high levels of 14,15-epoxyeicosatrienoic acid (14,15-EET). The study further proposed that CYP4Z1 may promote the tumourgenesis by a mechanism other than the direct production of 20-HETE^15^.

CYP1B1 plays an important role in the metabolism of many endogenous and exogenous compounds, including numerous carcinogens^16^. CYP1B1 was reportedly found to oxidize endogenous compounds such as steroids specially estrogen and melatonin. Similar fashion of metabolism was also demonstrated with exogenous compounds such as aromatic amines and polycyclic aromatic hydrocarbons converting them to active carcinogenic metabolites^17^. It is an extrahepatic enzyme where it is selectively expressed in many tumours, compared to undetectable expression in neighbouring normal cells. Elevated protein expression was detected in several tumours of the brain, breast, colon, ovary, and prostate^18–24^. Moreover, high expression was found in metastatic tumours^21^. This expression was associated with the tumour stage and grade of the disease^25^. Despite mRNA expression in several healthy tissues, its corresponding protein expression was rarely characterized^18,26^. In cell line models, CYP1B1 was highly expressed and its expression enhanced cell invasion and proliferation, and hampered cell apoptosis^25,27^. Additionally, CYP1B1 overexpression reduced the sensitivity of cancer cells to anti-cancer drugs such as paclitaxel and docetaxel and induced drug resistance^28,29^. Therefore, the differential and tumour selective expression of CYP1B1 represents a novel opportunity for the development of new anticancer therapeutic strategies.

Considering the fact that, and until the date of this paper, there are no prior studies assessing the expression of CYP4Z1 and CYP1B1 in bladder cancers, this study aims to examine the aberrant expression of CYP4Z1 and CYP1B1 in different subtypes of bladder cancer and correlate their expressions with baseline demographic and clinicopathologic features.

## Materials and methods

### Tissue specimens

A panel of Formalin-fixed, paraffin-embedded specimens were collected from the pathology department, King Hussein Medical Hospital, Royal Medical Services, Amman, and King Abdullah University Hospital, Irbed, Jordan. This panel consisted of 160 urothelial carcinomas, 16 squamous cell carcinomas, seven papillary adenocarcinomas, nine mucinous adenocarcinomas, eight adjacent normal bladder tissues, and eight normal bladder tissues. Patients who underwent preoperative chemotherapy or radiotherapy were excluded from the study. Information regarding age, tumour histopathology and the stage of the disease was extracted from the patients’ files. All personal data for samples were kept anonymous. The study was performed after approval of the local research and ethics committee in the Faculty of Medicine, University of Mutah. Informed consent was obtained from all participants or their legal guardians before starting the study. The study was conducted according to regulations set by the Declaration of Helsinki in 2013.

### Immunohistochemistry

Five archived µm-thick Formalin-Fixed and Paraffin-Embedded (FFPE) tissue sections were de-waxed in xylene and rehydrated to water through serial dilutions of alcohol. Endogenous peroxidase activity was blocked with freshly prepared 1% hydrogen peroxide for 30 min at room temperature, followed by washing in PBS. Sections then underwent the antigen retrieval process by being microwaved at 650 W in ten mM citrate buffer, pH 6.0, for 20 min. Following a wash in PBS, the non-specific binding sites for antibodies were blocked with 2.5% normal goat serum for 20 min at room temperature in a humidified chamber. For CYP4Z1 immunodetection, sections were covered with rabbit polyclonal antibody specific for CYP4Z1 (STJ92604) (St John's Laboratory Ltd, UK) at a concentration of 5 μg/ml overnight at 4C°. For CYP1B1 immunodetection, sections were covered with rabbit polyclonal antibody specific for CYP1B1 (NBP2-24722) (Novus Biological, USA) at a concentration of 10 μg/ml for one hour at room temperature. Sections were then washed in PBS and incubated with ImmPRESS (Peroxidase) polymer goat anti-rabbit IgG reagent for 30 min at room temperature (MP-7451, Vector Laboratories, Burlingame, USA). Immunoreactivity was visualised by covering sections with 3,39-diaminobenzidine tetrahydrochloridechromogen (DAB) (Vector Laboratories Ltd, Peterborough, UK) solution for 2–5 min. Following colour development, sections were then counterstained in Harris’s haematoxylin solution, dehydrated, and mounted with coverslips using a dibutylphthalate polystyrene xylene (DPX) medium. The negative control samples (procedural controls) were prepared by omitting the primary antibody to examine the secondary antibody binding.

### Scoring

Expression of CYP4Z1 and CYP1B1 was assessed according to the previously described semi-quantitative methods^30^. Evaluation of histologic slides was manually carried out by two independent pathologists. The criteria for CYP4Z1 and CYP1B1 positivity were based on the percentage of tumour cells showing expression. The cells were considered positive for CYP4Z1 expression if they demonstrated clear membranous or cytoplasmic immunoreactivity. For CYP1B1, cells were considered positive if they demonstrated cytoplasmic immunoreactivity. The scoring results were presented as negative (0), low (1), intermediate (2) and high (3). The score ‘negative’ indicated an absence of expression. A score of ‘low’ was applied to cells that had an expression of less than 33%. Sections were allocated a score of ‘intermediate’ when they had an expression of 33–66% of the cells, whilst a ‘high’ score indicated an expression in more than 67% of the cells.

### Statistical analysis

The software of SPSS version-19 (Statistical Packages for Social Sciences) was used to analyze the statistical data. Data were expressed in simple measures of frequency and percentage. Pearson’s Chi-square test (2-test) with the function of an ANOVA test or Kruskal–Wallis test was used for discrete variables to assess differences in expression. *p* < 0.05 was considered statistically significant.

### Results

#### Baseline demographic and clinicopathologic features

All the demographic and clinical data were available for 208 cases, where 192 were diagnosed with bladder cancer and the remaining 16 presented a normal pathology (Table 1 and Table 2). The mean age for patients was estimated at 58.9 ± 12.7 years. The most common bladder cancer subtype among the patients enrolled in this study was urothelial carcinoma (160 cases, 76.9%). Other subtypes included 16 squamous cell carcinomas (7.7%), nine mucinous adenocarcinomas (4.3%), and seven papillary adenocarcinomas (3.4%). Most of the bladder cancer cases were of histological grade II (82 cases, 42.7%), whereas grade I constituted 34.4% (66 cases) and grade III 22.9% (44 cases). Regarding the histological stage, more than half of the cases were stage 2 (101 cases, 52.6%), while the other cases distributed between the other stages as follow; stage 1 (53, 27.6%), stage 3 (34, 17.7%) and stage 4 (4, 2.1%). Moreover, more than half of the cases were found to invade the muscularis propria (101, 52.6%), whereas 19.5% of the cases were found to be invading through the muscularis propria into the subserosa or the perirectal tissues. Lymph node metastasis was only detected in three cases (1.5%).

**Table 1:** Association between CYP4Z1 expression and clinicopathologic features.

Characteristic	CYP4Z1-positive n = 113 (54.3%)	CYP4Z1-negative n = 95 (45.7%)	*P* value
**Gender**			0.373
Male (n = 166, 79.8%)	94 (56.6%)	72 (43.4%)	
Female (n = 42, 20.2%)	22 (52.4%)	20 (47.6%)	
**Histological type**			0.776
Urothelial carcinoma (n = 160, 76.9%)	91 (56.9%)	69 (43.1%)	
Squamous cell carcinoma (n = 16, 7.7%)	10 (62.5%)	6 (37.5%)	
Mucinous adenocarcinoma (n = 9, 4.3%)	5 (55.6%)	4 (44.4%)	
Papillary adenocarcinoma (n = 7, 3.4%)	3 (42.9%)	4 (57.1%)	
Normal (n = 16, 7.7%)	4 (25%)	12 (75%)	
**Histological grade**			
I (n = 67, 34.4%)	25 (37.9%)	5 (62.1%)	0.001
II (n = 81, 42.7%)	56 (68.3%)	26 (31.7%)	
III (n = 44, 22.9%)	28 (63.6%)	16 (36.4%)	
Normal (n = 16, 7.7%)	4 (25%)	12 (75%)	
**Histological stage**			
1 (n = 53, 27.6%)	37 (69.8%)	16 (30.2%)	0.035
2 (n = 101, 52.6%)	47 (46.5%)	54 (53.5%)	
3 (n = 34, 17.7%)	22 (64.7%)	12 (35.3%)	
4 (n = 4, 2.1%)	3 (75.0%)	1 (25%)	
Normal (n = 16, 7.7%)	4 (25%)	12 (75%)	
**TNM staging**			0.043
T1N0M0 (n = 54, 28.1%)	37 (68.5%)	17 (31.5%)	
T2N0M0 (n = 101, 52.6%)	47 (46.5%)	54 (53.5%)	
T3N0M0 (n = 34, 17.7%)	22 (64.7%)	12 (35.3%)	
T3N1M0 (n = 2, 1.0%)	2 (100%)	0 (0%)	
T3N2M0 (n = 1, 0.5%)	1 (100%)	0 (0%)	
Normal (n = 16, 7.7%)	4 (25%)	12 (75%)

**Table 2:** Association between CYP1B1 expression and clinicopathologic features.

Characteristic	CYP1B1-positive n = 160 (76.9%)	CYP1B1-negative n = 48 (23.1%)	*P* value
**Gender**			0.223
Male (n = 166, 79.8%)	131 (78.9%)	35 (21.1%)	
Female (n = 42, 20.2%)	36 (85.7%)	6 (14.3%)	
**Histological type**			0.002
Urothelial carcinoma (n = 160, 76.9%)	130 (81.2%)	30 (18.8%)	
Squamous cell carcinoma (n = 16, 7.7%)	14 (87.5%)	2 (12.5%)	
Mucinous adenocarcinoma (n = 9, 4.3%)	8 (88.9%)	1 (11.1%)	
Papillary adenocarcinoma (n = 7, 3.4%)	5 (71.4%)	2 (28.6%)	
Normal (n = 16, 7.7%)	3 (18.7%)	13 (81.3%)	
**Histological grade**			
I (n = 67, 34.4%)	53 (79.1%)	14 (20.9%)	0.003
II (n = 81, 42.7%)	68 (84.0%)	13 (16%)	
III (n = 44, 22.9%)	36 (81.8%)	8 (18.2%)	
Normal (n = 16, 7.7%)	3 (18.7%)	13 (81.3%)	
**Histological stage**			
1 (n = 53, 27.6%)	45 (84.9%)	8 (15.1%)	0.002
2 (n = 101, 52.6%)	74 (73.3%)	27 (26.7%)	
3 (n = 34, 17.7%)	34 (100%)	0 (0%)	
4 (n = 4, 2.1%)	4 (100%)	0 (0%)	
Normal (n = 16, 7.7%)	3 (18.7%)	13 (81.3%)	
**TNM staging**			0.011
T1N0M0 (n = 54, 28.1%)	47 (85.5%)	11 (14.5%)	
T2N0M0 (n = 101, 52.6%)	74 (73.3%)	27 (26.7%)	
T3N0M0 (n = 34, 17.7%)	34 (100%)	0 (0%)	
T3N1M0 (n = 2, 1.0%)	2 (100%)	0 (0%)	
T3N2M0 (n = 1, 0.5%)	1 (100%)	0 (0%)	
Normal (n = 16, 7.7%)	3 (18.7%)	13 (81.3%)	

#### CYP4Z1 expression and its relation to demographic and clinicopathologic features

**Figure 1:**
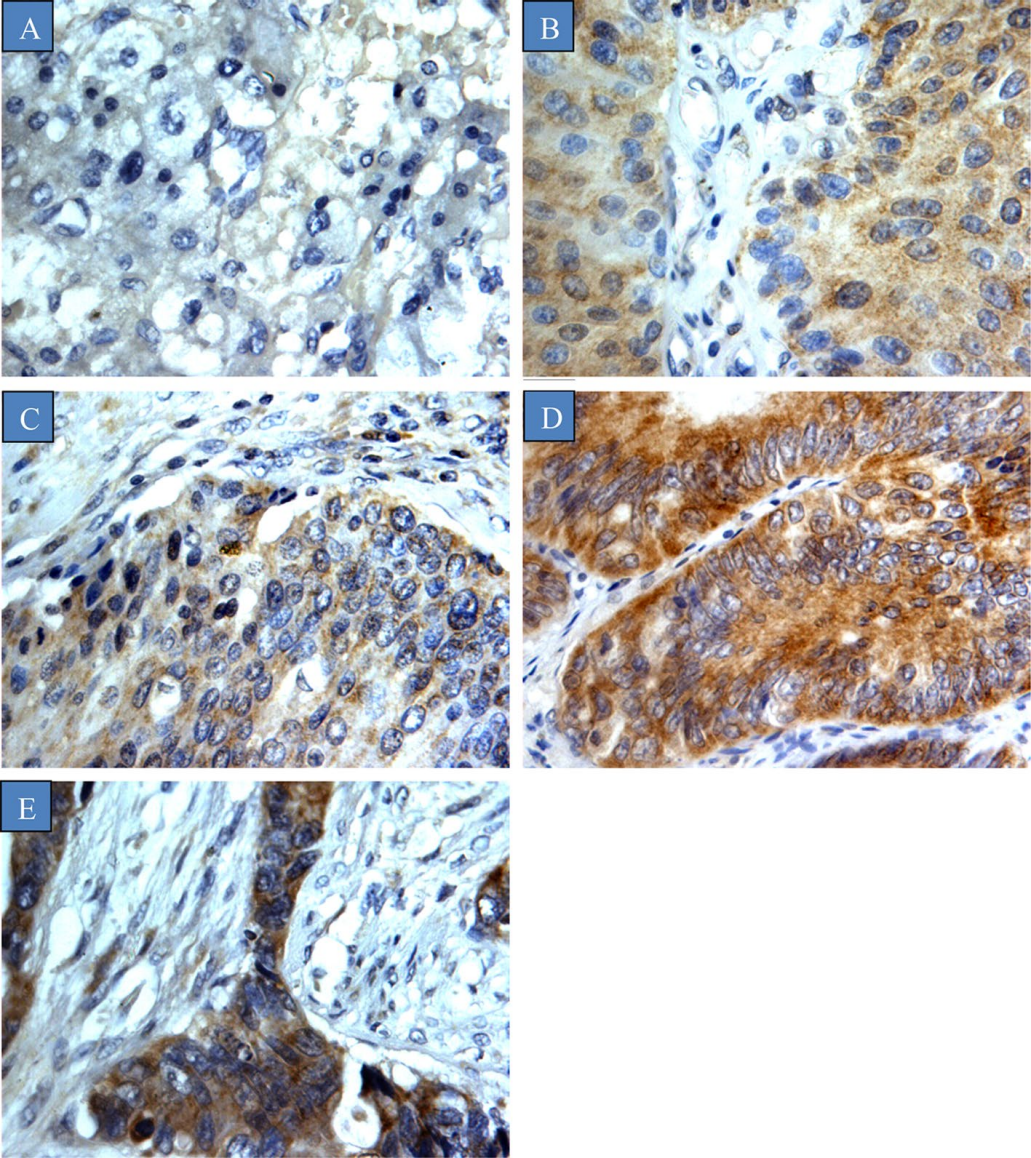
CYP4Z1 expression in different types of bladder cancers. Tumours were classified on the bases of histological type. (**A**) Normal bladder tissue, (**B**) Urothelial carcinoma, (**C**) Squamous cell carcinoma, (**D**) Papillary adenocarcinoma, and (**E**) Mucinous adenocarcinoma.

The relationship between CYP4Z1 expression in bladder cancers and various clinicopathologic features is shown in Table 1. CYP4Z1 expression was predominantly localized to the membrane or cytoplasm of the cells without any considerable staining noted in the nuclei (Fig. 1). CYP4Z1 was expressed in 113 (54.3%) cases of bladder cancers, whereas others demonstrated no expression (95, 45.7%). Weak CYP4Z1expression was demonstrated in four (25%) cases of normal tissues. To investigate the relationship between CYP4Z1expression and various patient features, the patients were grouped according to the status of CYP4Z1 (CYP4Z1-positive and negative). There was no significant difference in CYP4Z1expression among male and female patients; CYP4Z1expression was observed in 56.6% (94 cases) males and 52.4% (22 cases) females.

According to the pathological subtype of tumour, CYP4Z1 was strongly expressed in 91 (56.9%) cases of urothelial carcinoma, ten (62.5%) cases of squamous cell carcinoma, five (55.6%) cases of mucinous adenocarcinoma and three (42.9%) cases of papillary adenocarcinoma. However, no statistical difference in CYP4Z1 expression across different pathological subtypes of tumour was observed. When the CYP4Z1 expression in different groups was compared, a significant difference was identified between corresponding normal tissues and urothelial carcinoma, as well as papillary adenocarcinoma (*p* value < 0.05).

There was a significant association between the CYP4Z1 expression and histological grade. CYP4Z1 was more frequently expressed in grade II (56, 68.3%) and grade III (28, 63.6%) tumours, whereas grade I tumours demonstrated expression in only 37.9% of cases (*p* value < 0.05). Regarding the histological stage of the tumour, a significantly higher rate of CYP4Z1 expression was observed in the advanced stage of the disease (stage 3 64.7%, and stage 4 75.0%) compared with the early stages of the disease. Additionally, CYP4Z1 was significantly expressed across all clinical TNM stages, although it was more obvious in patients with lymph node metastasis (*p* value < 0.05).

#### CYP1B1 expression and its relation to demographic and clinicopathologic features

**Figure 2:**
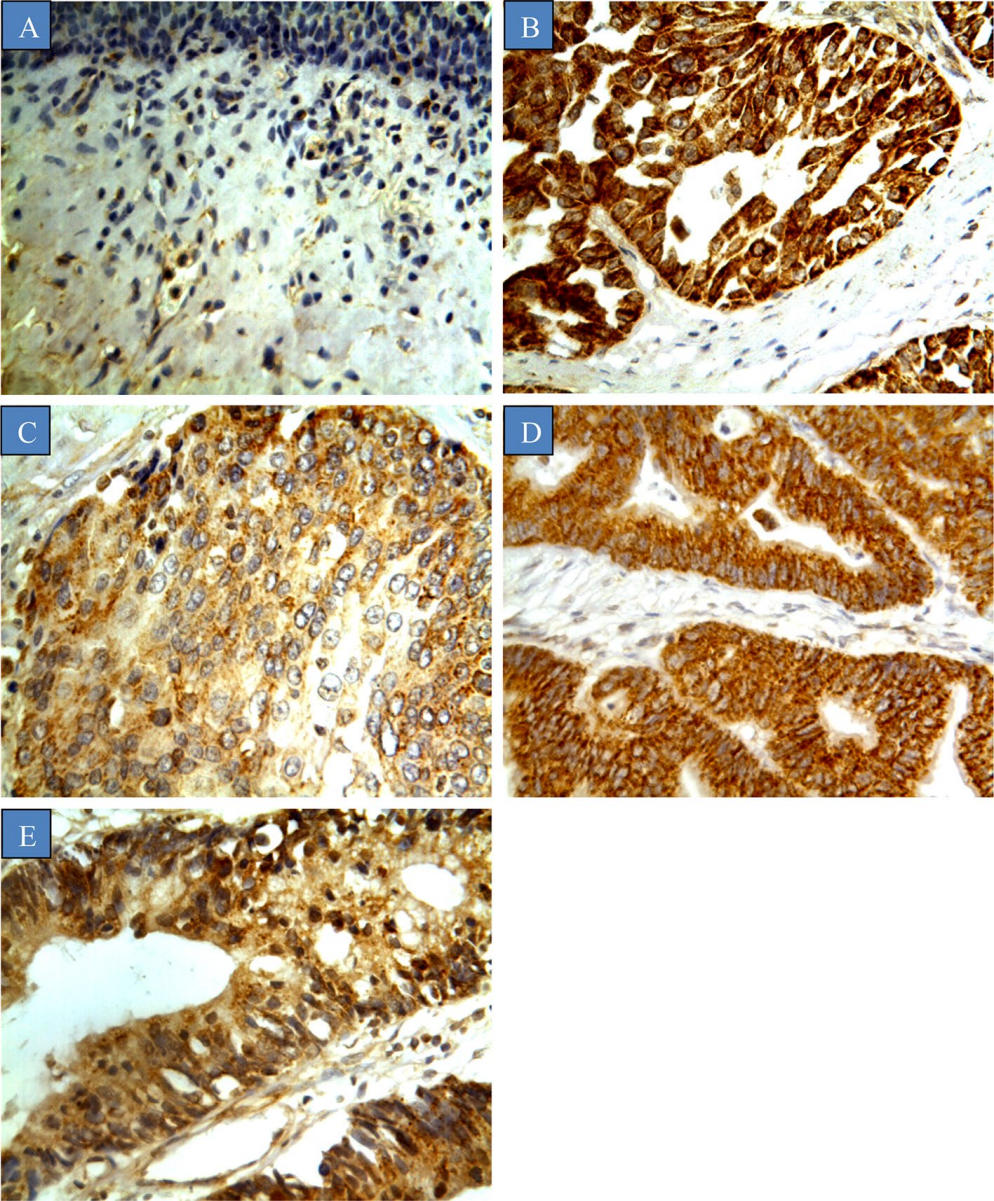
CYP1B1 expression in different types of bladder cancers. Tumours were classified on the bases of histological type. (**A**) Normal bladder tissue, (**B**) Urothelial carcinoma, (**C**) Squamous cell carcinoma, (**D**) Papillary adenocarcinoma, and (**E**) Mucinous adenocarcinoma.

The relationship between CYP1B1 expression in bladder cancers and various clinicopathologic features is shown in Table 2. CYP1B1 was highly expressed in the majority of bladder cancer samples (76.9%), while normal samples exhibited weak to undetectable expression (18.7%) (Fig. 2). CYP1B1 immunoreactivity was predominately displayed in the cytoplasm of the cells without any staining exhibited in the membranes and nuclei. Moreover, there was no obvious intratumour heterogeneity in staining observed across all the samples stained. To examine the relationships between various patient features and CYP1B1 expression, patient features were stratified according to the status of CYP1B1expression (CYP1B1-positive and negative). CYP1B1 was expressed in 131 (78.9%) males and 36 (85.7%) females.

A significant difference in CYP1B1 expression between different pathological sutypes of bladder cancers and corresponding normal samples was observed (*p* value < 0.05). CYP1B1 was strongly expressed in mucinous adenocarcinoma (88.9%), squamous cell carcinoma (87.5%), urothelial carcinoma (81.2%), and papillary adenocarcinoma (71.4%). Moreover, there was a significant association between histological stage, and TNM staging (*p* value < 0.05). CYP1B1 expression was identified across all tumour grades (grade I 79.1%, grade II 84.0% and grade III 81.8%). Likewise, CYP1B1 was markedly expressed in advanced stages of the disease (stage 3 100%, and stage 4 100%) as opposed to patients with the early stages (stage 1 84.9%, and stage 73.3%). In addition, CYP1B1 expression was positively correlated with TNM staging, where the expression was higher in stages with lymph node metastasis.

## Discussion

The current study investigated the CYP4Z1 and CYP1B1 expressions across a panel of different types of bladder cancer to confirm the hypothesis that CYP4Z1and CYP1B1 expressions may present novel opportunities for the development of new treatments for bladder cancer. Although many studies demonstrated the aberrant expression of CYP4Z1 in many tumours, including in the breast, ovary and prostate the CYP4Z1 enzyme association with other tumours remains at the forefront of current research^5–10,31^. Our study is the first to present convincing evidence of strong CYP4Z1 expression in different pathological subtypes of bladder cancer compared with low expression in corresponding normal tissues. Despite the small number of clinical samples other than urothelial carcinoma, some significant correlations were evident, particularly between papillary adenocarcinoma and the corresponding normal tissues.

Our results are concordant with previous studies demonstrating high CYP4Z1 expression in tumours of the breast, ovary and prostate compared to normal tissues^5–9,13,31^. Additionally, a strong over-expression of CYP4Z1 was seen in metastatic tumour samples compared with primary tumours^7,9^. This over-expression was associated with poor prognosis and was proposed to be an independent biomarker for prostate and ovarian cancers^7,8^. Since our clinical samples did not include any metastatic tissues for bladder cancer, further studies are needed, including studies on clinical metastatic tissues in bladder cancer.

Several studies have linked the CYP4Z1 expression with clinicopathologic features of different cancer patients. In this study, some significant associations were possible between CYP4Z1 expression and clinicopathological features. CYP4Z1 was expressed in different tumour grades, but the expression increased gradually with the increasing of tumour grade. This is consistent with other studies showing this kind of association, where CYP4Z1expression was correlated with increasing tumour grades^5,9^. Moreover, strong CYP4Z1expression was observed in advanced stages of the disease compared with low stages. Additionally, an interesting trend of high CYP4Z1expression was apparent in patients with lymph node metastasis. These results highlight the potential role of the CYP4Z1 enzyme in bladder cancer malignancy.

Several reports indicated high CYP1B1 expression in different tumours and a lack of expression in normal tissues, particularly hormone-responsive tumours such as breast, ovary, and prostate^19,21,23,24^. However, our study is the first to report high CYP1B1 expression in a large panel of bladder cancers, as opposed to corresponding normal tissues. This is consistent with a previous pilot study that demonstrated CYP1B1 expression in a smaller sample of bladder cancer patients (eight cases)^18^. Moreover, an increased CYP1B1 expression was also observed in metastatic ovarian cancers compared with primary tumours and normal tissues^21^. The expression profile of CYP1B1 is suggestive to have a potential role as a biomarker and target for anti-cancer therapy for renal cell carcinomas and ovarian cancers^25,28^.

Importantly, CYP1B1 expression was strongly associated with the histological stage of the disease. CYP1B1 expression was markedly higher in patients with advanced stages of the disease than those at the early stages of the disease. Concordant with previous studies, such an association was reported where CYP1B1 was markedly expressed in late clinical stages of ovarian cancers and renal cell carcinomas^25,28^. Despite the small number of patients with lymph node metastasis, a clear trend of high CYP1B1expression was apparent in patients with advanced TNM stages of lymph node metastasis. Similarly, high CYP1B1expression was demonstrated in metastatic samples of ovarian cancer patients^21^. Taken together, these results propose that the CYP1B1 enzyme might be a driver in tumour malignancy and it, therefore, represents a tumour biomarker and a potential target for the development of new cancer therapeutic strategies.

## Conclusion

To the best of our knowledge, our present study is the first report identifying the expression of CYP4Z1 and CYP1B1 in a large panel of bladder cancer. CYP4Z1 and CYP1B1 proteins are expressed at much higher rate in bladder cancer than in corresponding normal tissues. Significant associations were possible between the unique expression of CYP4Z1 and CYP1B1 and clinicopathological features. Both enzymes were strongly expressed in advanced stages of the disease. These results reinforce previous reports and strongly support CYP4Z1 and CYP1B1 as attractive targets for the development of novel cancer therapies. Further studies are required to look deeply into the mechanistic role of CYP4Z1 and CYP1B1 in tumour progression and to better validate whether they are druggable targets.

## Abbreviations

CYP: Cytochrome P450
CYP4Z1: Cytochrome 4Z1
CYP1B1: Cytochrome 1B1
20-HETE: 20-Hydroxyeicosatetraenoic acid
FFPE: Formalin-Fixed and Paraffin-Embedded (FFPE)
DAB: 3,39-Diaminobenzidine tetrahydrochloride chromogen
DPX: Dibutylphthalate polystyrene xylene
